# Integrative Oncology and the Clinical Care Network: Challenges and Opportunities

**DOI:** 10.3390/jcm12123946

**Published:** 2023-06-09

**Authors:** George Semeniuk, Bahareh Bahadini, Eugene Ahn, Jasmine Zain, Jessica Cheng, Ameish Govindarajan, Judy Rose, Richard T. Lee

**Affiliations:** 1 City of Hope Comprehensive Cancer Center, Duarte, CA 91010, USA; gsemeniuk@coh.org (G.S.); bbahadini@coh.org (B.B.); eugene.ahn@ctca-hope.com (E.A.); jazain@coh.org (J.Z.); jescheng@coh.org (J.C.); agovindarajan@coh.org (A.G.); jrose@coh.org (J.R.); 2 City of Hope Chicago, Zion, IL 60099, USA

**Keywords:** integrative oncology, clinical care network, implementation

## Abstract

Integrative oncology is a new and growing field of cancer care. Integrative oncology is a patient-centered, evidence-based field of comprehensive cancer care that utilizes integrative therapies such as mind-body practices, acupuncture, massage, music therapy, nutrition, and exercise in collaboration with conventional cancer treatments. Patient interest and utilization has been growing over the past two decades. Clinical research has shown the benefits of these approaches to improving symptom management and quality of life, and is now being incorporated into national guidelines from the National Comprehensive Cancer Network (NCCN) and American Society for Clinical Oncology (ASCO). The availability of these services at cancer centers is growing, although the structure and implementation of integrative oncology remains highly variable. This article discusses the benefits of integrative oncology and provides an overview of the current state of integrative oncology programs nationwide. Current challenges and opportunities for cancer centers to provide integrative services is reviewed in the areas of programmatic structure, clinical service, education, and research.

## 1. Introduction

**Figure 1:**
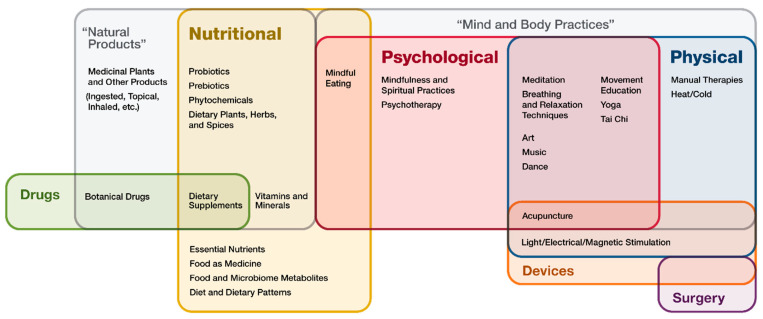
NCCIH ICAM approaches.

Over 19 million people around the world were diagnosed with cancer and almost 10 million died from cancer in 2020 [1]. By 2040, new cases and death totals are expected to reach approximately 28 million and 16 million, respectively [2]. Cancer treatment alone costs the world approximately USD 1.2 trillion dollars annually. According to the National Center for Complementary and Integrative Health (NCCIH), “if a non-mainstream approach is used together with conventional medicine, it’s considered complimentary”, while “if a non-mainstream approach is used in place of conventional medicine, it’s considered alternative” [3]. Therefore, integrative medicine is practiced in combination with conventional cancer care, not as an “alternative”. Thus, these types of therapies are commonly termed integrative, complementary, and alternative medicine (ICAM) and have become increasingly popular in Western medicine. The Academic Consortium for Integrative Medicine and Health describes this approach as one that “reaffirms the importance of the relationship between practitioner and patient, focuses on the whole person, is informed by evidence, and makes use of all appropriate therapeutic and lifestyle approaches, healthcare professionals and disciplines to achieve optimal health and healing”. The NCCIH divides ICAM modalities into three main categories: nutrition, psychological, and physical. Nutrition approaches focus on food as medicine and encompass special diets, dietary patterns, and natural products (i.e., dietary supplements such as vitamins, minerals, herbs, and botanicals). Psychological aspects include mindfulness and spirituality, while physical modalities include massage and spinal manipulation, and increasingly popular is the combination of psychological and physical approaches known as mind and body practices, including acupuncture, massage therapy, mindfulness, meditation, music therapy, and yoga (see Figure 1 from NCCIH) [3].

As this applies to cancer care, The Society of Integrative Oncology (SIO) defines integrative medicine as a “patient centered, evidence-informed field of cancer care that utilizes mind and body practices, natural products, and/or lifestyle modifications from different traditions alongside conventional cancer treatments” [4]. In practice, integrative medicine is a multidisciplinary approach to treating patients with efforts to combine the benefits of integrative and conventional modalities that have been shown to be safe and effective. These types of therapies are provided by a different group of professional caregivers, with different educational backgrounds, credentials, and regulatory oversight that varies by therapy, geography, and culture.

The use of ICAM has become increasingly popular. According to the 2012 National Health Interview Survey, 59 million Americans over the age of four used at least one complementary health approach, equating to USD 30.2 billion dollars in out-of-pocket expenses [5]. Dietary supplements and yoga appeared to be the most popular modalities in 2017, with 57.6% and 14.2% of adults reporting their usage, respectively [6,7]. People use integrative medicine for a wide array of diseases and conditions. According to surveys, a majority of patients use integrative medicine most commonly for back pain or back problems, head or chest colds, neck pain or neck problems, joint pain or stiffness, and anxiety or depression [8]. As more patients are using integrative medicine, so are practitioners. According to a national survey of medical oncologists, one third reported using ICAM and nearly two thirds had recommended some form of ICAM to patients [9].

Cancer patients and survivors in particular, increasingly look to ICAM in attempt to decrease recurrence, manage the side effects of treatment, and manage and treat other comorbidities [10]. More specifically, approximately one third of cancer patients reported using ICAM in the last 12 months in 2019, with herbal supplements being the most commonly used modality. Of the cancer patients using ICAM, 29.3% did not disclose their ICAM use to their physicians [11]. This high use of herbal supplements, in combination with lack of disclosure to their healthcare providers, is worrisome not only because dietary supplements are not thoroughly regulated, and thus may have quality and safety issues, but also because many have drug interactions and the potential for negative outcomes. Therefore, it is important for physicians to discuss ICAM with their patients to ensure they are using ICAM in safe and effective ways. This also requires educating physicians on the various ICAM modalities, such as their safety, efficacy, and effects on the body and on other conventional treatments.

Despite the growing efforts to provide conventional cancer care globally, and similar attempts to increase the use of ICAM into Western medicine practices, a systematic integration of these two approaches is still missing. This is where the field of integrative oncology can serve as a bridge between the two models and help both patients and practitioners find solutions to navigate between the two systems. This article focuses on integrative oncology and the current state of practice, with an emphasis on clinical care network as most patients are treated at community centers near home rather than large academic cancer centers.

## 2. Benefits of Integrative Oncology

Growing evidence indicates the important role of integrative oncology on outcomes in cancer care, with most studies indicating improvement in symptoms and quality of life including pain, nausea/vomiting, anxiety, hot flashes, insomnia, neuropathy, and dry mouth [12]. As a result, integrative therapies are now being incorporated in national guidelines from the National Comprehensive Cancer Network and the American Society for Clinical Oncology [13,14,15].

The mind-body connection is an important aspect of integrative oncology, as emphasized in the recent Institute of Medicine (IOM) report “Cancer Care for the Whole Patient” (IOM) [16]. In this comprehensive report, it is mentioned that “cancer care today often provides state-of-the-science biomedical treatment but fails to address the psychological and social (psychosocial) problems associated with the illness. These problems—including… anxiety, depression or other emotional problems—cause additional suffering, weaken adherence to prescribed treatments, and threaten patients’ return to health”. Extensive research has documented that mind–body interventions appear to address many of the issues mentioned in the IOM report.

Mindfulness meditation is the practice of bringing one’s attention to the present via thoughts, feelings, emotions, and physical presence with openness. One of the most well studied techniques is Mindfulness-Based Stress Reduction (MSBR), which can be taught via an 8-week course [17,18], and a variety of other techniques have also been studied such as yoga meditation and Tibetan meditation. The MBSR training is generally a 2.5 h session each week for 8 weeks, along with an 8 h retreat. A clinical trial of 229 women found that MSBR significantly improved total mood, quality of life, and well-being compared to the control group, in women who had undergone breast cancer treatment [19]. Another randomized controlled trial examining breast cancer survivors found mindfulness awareness practices to significantly reduce stress, pro-inflammatory gene expression, inflammatory signaling, fatigue, sleep disturbance, and vasomotor symptoms, though these effects, aside from cancer-related distress, did not persist at a 3 month follow-up [20]. Six other studies found that meditation can also significantly decrease anxiety, fatigue, and depression in cancer patients [21,22,23,24,25,26], as well as lead to stress reduction and improve overall quality of life [21,27]. Anderson et al. demonstrated a survival advantage among breast cancer patients randomized to a psychological intervention with more than 10 years of follow-up [28]. Although the exact mechanism behind this effect remains unclear, the authors have proposed several theories. Psychological therapies such as Cognitive Emotional Behavior Therapy (CEBT), Cognitive Behavioral Therapy (CBT), and supportive-expressive therapy aim to alleviate anxiety and depression commonly associated with cancer treatments. These negative emotional states are believed to suppress the immune and neuroendocrine systems, which may impact survival [29]. Patients are taught problem-solving skills, cognitive flexibility, and relaxation techniques to better cope with stressful situations. However, a recent meta-analysis of psychosocial interventions showed only minimal short-term improvements in survival, with individually delivered interventions failing to show any survival benefits [30]. ASCO/SIO guidelines recommend meditation to reduce anxiety (Grade A), treat mood disturbance and depressive symptoms (Grade A), and improve quality of life (Grade A) [31].

Another important mind–body therapy has been yoga. One meta-analysis of randomized controlled trials found that yoga significantly decreased depression, distress, and stress in cancer patients, though the number of studies analyzed was limited to ten, which varied in quality [32]. Another review of thirteen randomized controlled trials found yoga to improve cancer patients’ mental health, quality of life, and treatment-related side effects, while decreasing stress, anxiety, and depression [33], which supports a review by Greenlee et al. that also found an improvement in quality of life and decrease in distress, anxiety, and depression in cancer patients that practiced yoga [21]. Similarly, Cramer et al.’s meta-analysis of 23 randomized controlled trials found that yoga can improve quality of life and decrease fatigue and sleep disturbances in the short-term, but did not affect depression or anxiety [34]. ASCO/SIO guidelines recommend yoga to reduce anxiety (Grade B), improve mood disturbance and depressive symptoms (Grade B), and improve quality of life (Grade B). They also state that yoga can be considered for post-treatment fatigue (Grade C) and that gentle yoga can be considered to improve sleep (Grade C) [31].

Acupuncture is a modality that has gained increasing acceptance due to the growing clinical research demonstrating its positive outcomes. Its main benefits for cancer patients surround symptom management, though the outcomes are dependent on the symptom. For example, a randomized controlled trial consisting of 226 women with early-stage breast cancer showed a statistically significant reduction in aromatase inhibitor-related joint pain in patients that received 12 acupuncture sessions over 6 weeks, compared to the women who received sham acupuncture, or no acupuncture [35]. Another large randomized clinical trial by Shen et al. focused on 104 women with high-risk breast cancer and found electroacupuncture to significantly decrease the number of emesis episodes compared to minimal needling or antiemetic pharmacology after 5 days, but these differences disappated by day 9 [36]. Additionally, four reviews found that acupuncture reduces cancer-related pain [37,38,39,40], while two other reviews found not enough statistically significant evidence to draw this conclusion [41,42], and a review by Paley et al. found that acupuncture’s effect on pain management varied by cancer type [43]. A meta-analysis by Tao et al. found that acupuncture can also increase quality of life and reduce symptoms such as pain, fatigue, sleep disturbance, and gastrointestinal discomfort [39], while two reviews found decreases in fatigue, chemotherapy-induced nausea and vomiting, and leukopenia [21,42]. The recent SIO-ASCO joint guidelines on integrative therapies for cancer pain found evidence to support the use of acupuncture aromatase inhibitor-related joint pain, general cancer pain and musculoskeletal pain [15].

Massage is another popular integrative therapy that has been evaluated for patients with cancer. A randomized trial of 380 patients with advanced cancer with moderate to severe pain found that six 30 min massage therapy sessions over 2 weeks can significantly reduce pain and mood compared to simple-touch sessions, while sustained pain, quality of life, symptom distress, and medication analgesics were not significantly different [44]. Meanwhile, other studies have shown that massage therapy can decrease pain, fatigue, anxiety, nausea, and depression from 42.9% to 59.9% in cancer patients, though the effects are relatively short-term [21,45,46,47,48]. Massage was recommended by the SIO-ASCO joint guidelines to help reduce pain during palliative and hospice care [15].

Integrative oncology also emphasizes the importance of nutrition and physical activity in the health of cancer patients, and has been correlated with improved clinical outcomes. The Women’s Intervention Study (WINS) and the Women’s Health Eating and Living (WHEL) have found improvement in clinical outcomes, such as recurrence rates and overall survival with nutrition and physical activity [49,50]. The American Institute for Cancer Research and the World Cancer Research Fund have created a combined report for guidelines regarding nutrition and physical activity to prevent cancer. The American Cancer Society, American College of Sport Medicine, and ASCO have published guidelines for those with cancer [51,52,53]. These guidelines generally emphasize regular exercise, a plant-based Mediterranean style diet, limiting risk factor such as alcohol, and maintaining a healthy body weight. Adherence to these guidelines has been associated with improved survival, such as in colon cancer, and ongoing clinical trials are evaluating this in a more prospective manner [54]. Integrative oncology programs should incorporate these guidelines for cancer prevention and survivorship for patients. 

It is important to remember that various integrative modalities can cause harm to patients with cancer if used inappropriately. For instance, herbs may interact with drugs, interfering with cancer treatments or compounding toxicity [55,56]. Quality control issues are a major concern with natural products and herbal supplements because of the potential for product substitutions or fillers, contamination, and inaccurate labeling [57]. Moreover, some treatments used by patients may not be covered by Medicare or insurance plans, leading to financial constraints for patients and their families. Additionally, patients may suffer from psychological distress due to unrealistic expectations regarding these treatments, especially when used as an alternative to standard of care.

## 3. Current State of Integrative Oncology

As patient interest has grown in integrative oncology, cancer centers are responding by providing more services, as indicated by one study [58]. From 2009 to 2016, comprehensive cancer centers were offering more services, including herb/supplement consultation (89–96%), meditation (89%), acupuncture (89%), yoga (87%), massage (84%), music therapy (82%), and physician integrative medicine consultation (60%) [59]. Unfortunately, the exact details of how these services are provided is lacking and thus much of this information is garnered by the authors first-hand understanding through colleagues and attending conferences. Based on our experience, how these services are provided varies significantly at different clinical centers. At large academic cancer centers, the integrative medicine programs are often housed within the department of medicine or family medicine, such as at the University of Arizona and University of Wisconsin. These programs may or may not have strong connections to the cancer center. Additionally, with this model, integrative medicine services are often not found within the same clinical space as the cancer center and thus patients receive them at a different location, which may be a distance away. Many patients seek integrative services through community practices that generally operate separately from the main medical center. Therefore, patients receive clinical care at more than one system and most commonly, these medical systems are not connected and thus have limited communication.

The practice of integrative medicine has natural overlap with several related services, including supportive/palliative care, pain medicine, psychology, psychiatry, spiritual care, rehabilitation therapies (e.g., physical therapy, occupational therapy, speech therapy), prevention, and survivorship. The relationship between these other key disciplines and integrative programs is highly variable. In some institutions, these programs are within the same administrative structure as supportive/palliative care such as at Memorial Sloan Kettering Cancer Center and MD Anderson Cancer Center. In other cancer centers, it may exist primarily as a separate clinical program (Duke and University of California San Francisco), wherein faculty have primary academic appointments in other departments and divisions. The leadership of integrative oncology programs also comes from a variety of different disciplines, including family medicine, internal medicine, oncology (medical, radiation, and surgical) psychology, psychiatry, and naturopathic physicians, among others. How these integrative medicine clinical programs collaborate with other related services within the same institution is so diverse that no general trend can be ascertained. In general, the optimal structure and relationship within a comprehensive cancer center remains unclear and is still being evaluated.

## 4. Challenges and Opportunities for Integrative Oncology

Since no optimal model has been clearly identified for integrative oncology within a cancer center, we will highlight the characteristics of successful long-term integrative oncology programs within four main areas: programmatic and financial structure, clinical service, education, and research. The integrative oncology program should have senior level support within the cancer center to ensure long term viability. Many examples of programs being started with philanthropy investment, only later to slowly disappear, illustrate a common story among integrative medicine/oncology programs, which speaks to the importance of institutional support, but also programmatic leadership. Having oncologists either leading or establishing strong partnerships is critical to understanding the nuances with modern cancer care, as well as developing trust among the cancer center staff by being an advocate from within. A series of guidelines have been developed by SIO and endorsed by ASCO for highlighting evidence-based integrative therapies in breast cancer and more recently, integrative approaches to cancer pain [14,15]. Additional practice guidelines are currently under development by SIO to facilitate the incorporation of integrative therapies in an evidence-based approach.

Integrative medicine is patient-specific and based on a patient’s goals, values, cultures, and philosophy of health. Practice guidelines are often generic and difficult to adapt to individual patients. A universal, one-size-fits-all approach to implementation of these evidence-based guidelines is impossible and therefore personalization and cultural tailoring are necessary for each cancer center. Additionally, the limited published data is available discussing how to establish integrative oncology programs. Based on our knowledge and experience, challenges to implementing a community network integrative oncology program include a lack of financial resources, clinical delivery due to limited numbers of integrative oncology trained practitioners and leaders, as well as difficulties advocating and collaborating with other departments (e.g., palliative care physicians and social workers), and developing an educational forum for disease-specific cancers.

Financial: Funding and budgetary constraints pose the number one challenge of a community integrative network program. Increased cancer survival rates, diminishing payments and reimbursements, and expanding and aging populations make obtaining enough funding to support a network expansion of an existing integrative program difficult. Without more rigorous scientific evidence in large, reproducible, randomized controlled studies, it will be difficult to justify the costs of payments from Medicare and insurers for many integrative supplements and practices. Most patients will have high out-of-pocket costs already limiting their access to standard care in addition to receiving integrative therapies, which has been identified in surveys of patients [60,61]. Developing a financially feasible integrative oncology program that expands patient access is challenging, but necessary. Insurance will typically cover integrative oncology visits with an MD or NP. Still, visits with other integrative care providers, such as naturopaths and acupuncturists, often incur high out-of-pocket costs. Additionally, racial disparities and lower socioeconomic patients are often under-represented in integrative oncology programs due to financial constraints, time away from work, transportation, and lack of childcare. Ideally, programs should offer a sliding scale fee structure that allows patients of limited financial resources to still be able to receive integrative therapies as medically indicated.

The financial model also needs to be constantly evaluated for sustainability, as philanthropy is unpredictable. Achieving defined budgetary support from the healthcare system is paramount as the integrative oncology program matures. The clinical services themselves also provide significant revenue in which to sustain the program, although this alone is rarely enough. Thus, successful programs often draw from all three sources of funding: institutional, philanthropy, and clinical revenue. Programs should also focus on the return on investment (ROI) for healthcare systems, which may come in the form of increased patient satisfaction, improved quality of life, decreased cost or healthcare utilization, and market differentiation. Thus, the benefit of integrative programs goes beyond the revenue it is able to generate, which is not always understood by senior leaders.

Clinical Services: Clinical delivery of programs also varies between centers. Three types of services are generally found: outpatient, inpatient, and group. Mostly commonly, these services include integrative oncology consultation, acupuncture, massage, mind–body medicine, music therapy, nutrition, and exercise counseling. Careful consideration should be made in the referral process and, if possible, adherence to routine procedures for referral to other services such as supportive/palliative care or physical therapy. Where to deliver care is also a major challenge, given many cancer centers have outgrown the available clinical space. Ideally, these services should be provided within the cancer center so they can be both accessible and visible to patients. Some programs have begun to deliver services simultaneously while patients receive chemotherapy in the infusion suites, which then obviates the need for separate space and appointment.

Many comprehensive cancer centers have associated integrative oncology programs with their cancer services. These programs are intricately linked in proximity to large academic centers. However, incorporating an integrative oncology program into multiple community network sites poses many challenges. The demand for integrative oncology is increasing, but having all the available integrative services for patients at each network site will be complex and problematic. Barriers to developing and expanding such a program to the community include financial limitations, lack of skilled and certified integrative practitioners, spatial constraints at each site, and adaptation of innovative technology.

Additionally, the development and expansion of integrative oncology programs to academic network sites depends on the availability of a skilled workforce. Currently, most oncologists do not have an integrative oncology background. Only a minority have received formal integrative medicine training and board certification and dedicated therapists who focus only on oncology patients are scarce. Recruiting enough experienced integrative providers that could provide care at these individual sites makes it challenging to meet the high demands of interested patients. Developing an integrative oncology program in the community also requires increasing square footage space, including dedicated exam rooms, quiet areas for services such as meditation, and procedure rooms for massage and acupuncture treatments. Most importantly, merging integrative therapies into a cancer patient’s care plan will require the endorsement and adoption of the whole care team, including medical, radiation, and surgical oncologists, which requires a culture change for most places as these clinicians may not be aware of the value of integrative therapies. This also entails making sure services are available for referral by the treatment team.

Currently in community practices, the most readily available integrative oncology care model entails the patient seeing a conventional oncologist while also being managed in parallel by an integrative medicine provider who may or may not have had formal oncology training. Obviously, there are risks with this arrangement, especially if there is no collaboration or discussion. It is recommended in these settings to ensure there is a dialogue between the oncologist and the integrative medicine provider, including the sharing of medical records to reduce the chances of serious drug interactions and use of contraindicated interventions. Through this dialogue, there is an opportunity for the oncologist to gauge their level of comfort with the integrative medicine provider, and ensure the integrative medicine provider is using similar evidence-based standards as the oncologist. Not only will this promote safety and increase the knowledge base of the integrative medicine provider and oncologist, but a constructive relationship will eventually lead to referrals from the integrative medicine provider as well due to the mutual respect. Likewise, such interactions will help the oncologist identify practices that are marketed as integrative but are actually alternative. Sometimes patients may choose to seek out alternative therapies while also receiving conventional cancer treatment, and patients are encouraged to at least inform their oncology team so they are aware of the potential risks, even if they do not agree with the combined approach. The optimal situation is one in which an established working relationship exists between the oncology team and the integrative practitioners, and includes a shared medical record system for enhanced communication. Additionally, oncologists should consider developing a network of preferred providers in preparation for when patients inquire about receiving integrative therapies.

Telehealth has been rapidly adopted across several health systems in the United States and around the world during the COVID-19 pandemic, and as patients are more isolated from family and community they attempt to explore strategies and interventions to successfully manage their symptoms, incorporate a healthy lifestyle, and improve their overall health. Telehealth has had its own unique opportunities and challenges in oncology. Considering that cancer patients more frequently use ICAM than the general population, to meet patient’s needs, integrative oncology programs have become more widely available in several cancer centers [58]. Despite more integrative oncology programs have become available in cancer centers around the nation, access to such programs is still limited to major medical centers. Even when a cancer program has an established integrative oncology program, access may be further limited due to time constraints and challenges with the coordination of care with multiple appointment requirements and geographic barriers. Adopting telemedicine in integrative oncology may therefore help reduce some of these challenges. According to one study at University of Texas MD Anderson, looking at this paradigm shift during the pandemic, telehealth integrative oncology consultations were provided to 509 new patients from 21 April 2020 to 21 October 2020 compared to 842 new patients in person during the same period in 2019 [62]. In this study, it was concluded that delivering integrative oncology consultations using telehealth is feasible and meets patient’s needs. These patients reported lower symptom burden and more concerns about lifestyle, herbs, and supplements. Additional studies have also shown the ability of telehealth to effectively deliver diet and exercise interventions for patients with cancer [63,64,65,66]. We believe that use of the telehealth platform to provide integrative oncology consultations is beneficial if in-person consultation is not feasible to counsel patients on healthy lifestyle and address any questions regarding natural products or other integrative modalities.

Another option to consider is a group medical visit (GMV) model to increase access to specialized integrative oncology care. This model has been tested successfully in other community settings such as UCSF Integrative Oncology [67]. GMV aims to present integrative oncology information to groups of individuals, not to serve as a support group, except for informal patient interactions. Patients are divided into one of three appropriate series: patients on active treatment or maintenance treatment of their specific cancer (tumor-specific), patients finished with treatment and in remission (tumor agnostic), and any cancer patient with metastatic disease on treatment (tumor agnostic). Each series consists of three meetings: session 1 covers nutrition and cancer, session 2 covers cannabis and other supplements, and session 3 covers acupuncture and stress reduction therapies. All three meetings last 2 h, including didactic content presented by the physician, time for patient questions, and individual consultations in a nearby room. Groups include up to eight patients, and each is allowed to bring one guest.

The program for GMV integrative oncology visits has been demonstrated previously to be financially viable. It is more efficient for a provider to bill for more patients receiving shorter individual consultations as part of the GMV than to spend that time in private, 1 h consultations. The revenue from group visits significantly exceeds the revenue potential when compared to the time spent on individual visits. When similar models were implemented in other communities, patient and provider satisfaction surveys were high and rated with scores indicating the enjoyment and high value of sessions. 

Education and Research: The lack of awareness of integrative therapies has been noted by both patients and clinicians and is a barrier to utilization [60]. Therefore, the success of any integrative oncology program relies on the ability to inform patients and clinicians about the potential benefits of these approaches. A strategy for clinicians is to provide experiential opportunities for them to receive clinical services such as acupuncture or meditation. The services could be provided as part of a routine meeting such as a department/division meeting or even an in-service meeting for nurses. Another important aspect of training is the development of specialists in integrative oncology, including medical oncology, radiation oncology, and surgical oncology as well as integrative therapists specializing in cancer–acupuncture, massage, and mediation. Given the growing demand by patients, and the lack of clinicians to staff this demand, it is essential that comprehensive cancer centers consider this part of the long-term mission. Along with the SIO, only four academic institutions provide formal integrative oncology training for healthcare providers: MD Anderson, Memorial Sloan-Kettering, University of Arizona, and the University of Michigan [12].

To successfully provide integrative oncology in the community, patients need to be aware of the services offered and have an easy way to access the program promptly. Education is vital to helping patients consider integrative therapies as part of their treatment. One strategy is to utilize current infrastructure to help educate patients. Many cancer centers are exploring the use of patient navigators familiar with all the services offered to assist the patient with reinforcing and detailing their goals and treatment plan. Having the patient navigator be familiar with available integrative services is a significant opportunity to engage patients, as the navigator facilitates timely appointments, obtains medical records, and completes pre-authorizations. Many patients already find and use outside resources to help them with this process. Third-party companies and concierge physician services are often hired to advocate for the patient by reviewing medical research on their behalf, helping them locate practitioners and clinical trials, and offering integrative solutions for their problems with discussions on supplements, medical cannabis, nutrition, and lifestyle changes.

Although there are a growing number of large, randomized clinical trials of integrative therapies, there continues to be a relatively weak foundation of positive trials in oncology to compel insurance companies of both the clinical and financial benefit to cover these services. Clinical programs should consider incorporating routine collection of patient outcomes to demonstrate the real-world value of integrative interventions. These should also be accompanied by some financial analysis as well, given many of these benefits may come in the form of decreased length of stay or decreased use of healthcare services such as emergency room visits [68]. If infrastructure and funding allows, integrative oncology programs should be providing patients the opportunity to participate in clinical trials to, in turn, help produce more critical data to understand how to best use these approaches to improve patient care.

## 5. Conclusions

Integrative oncology is growing in cancer care, both in patient interest as well as in the positive clinical impact it provides. Benefits of early integrative oncology support may include optimization of symptom management and improved quality of life, as well as a potentially enhanced ability to deliver chemotherapy due to the mitigation of side effects by integrative oncology approaches. The complexity of cancer treatment regimens and the potential of complementary and integrative care, to either enhance or interfere with treatment, underscores the need for more integrative oncology providers. It is important that all practitioners involved in the care of cancer patients have the knowledge and skills to recognize the benefits of integrative oncology. Cancer centers, both at comprehensive cancer centers as well as community network sites, should provide opportunities for patients to utilize these therapies as part of the treatment plan. Programs require support from senior leadership and integrative oncology programs, and need to be culturally sensitive to meet the unique needs of their patient population. A global commitment to research is critical to advancing evidence-based integrative oncology programs.

## Data Availability

Not applicable.
